# Identifying risk factors of anemia among women of reproductive age in Rwanda – a cross-sectional study using secondary data from the Rwanda demographic and health survey 2014/2015

**DOI:** 10.1186/s12889-019-8019-z

**Published:** 2019-12-11

**Authors:** Dieudonne Hakizimana, Marie Paul Nisingizwe, Jenae Logan, Rex Wong

**Affiliations:** 1 Department of Global Health Delivery, University of Global Health Equity, Kigali, Rwanda; 2 School of Population and Public Health, University of British Columbia, Vancouver, BC Canada; 3 School of Public Health, Yale University, New Haven, CT USA

**Keywords:** Anemia, Women of reproductive age, Malaria, Prevalence, Associated factors

## Abstract

**Abstract:**

**Background:**

Anemia among Women of Reproductive Age (WRA) continues to be among the major public health problems in many developing countries, including Rwanda, where it increased in prevalence between the 2015 and 2010 Rwanda Demographic and Health Survey (RDHS) reports. A thorough understanding of its risk factors is necessary to design better interventions. However, to the best of our knowledge, no study has been conducted in Rwanda on a nationally representative sample to assess factors associated with anemia among WRA. Accordingly, this study was conducted to address such gap.

**Methods:**

A quantitative, cross-sectional study was conducted using data from the RDHS 2014–2015. The study population consisted of 6680 WRA who were tested for anemia during the survey. Anemia was defined as having a hemoglobin level equal to or below 10.9 g/dl for a pregnant woman, and hemoglobin level equal to or below 11.9 g/dl for a non-pregnant woman. Pearson’s chi-squared test and multiple logistic regression were conducted for bivariate and multivariable analysis, respectively.

**Results:**

The prevalence of anemia among WRA was 19.2% (95% CI: 18.0–20.5). Four factors were found to be associated with lower odds of anemia, including being obese (OR: 0.61, 95% CI: 0.40–0.91), being in the rich category (OR: 0.74, 95% CI: 0.63–0.87), sleeping under a mosquito net (OR: 0.85, 95% CI: 0.74–0.98), and using hormonal contraceptives (OR: 0.61, 95% CI: 0.50–0.73). Five factors were associated with higher odds of anemia, including being underweight (OR: 1.39, 95% CI: 1.09–1.78), using an intrauterine device (OR: 1.98, 95% CI: 1.05–3.75), being separated or widowed (OR: 1.35, 95% CI: 1.09–1.67), and living in the Southern province (OR: 1.45, 95% CI: 1.11–1.89) or in the Eastern province (OR: 1.41, 95% CI: 1.06–1.88).

**Conclusion:**

Anemia continues to pose public health challenges; novel public health interventions should consider geographic variations in anemia risk, seek to improve women’s economic statuses, and strengthen iron supplementation especially for Intrauterine device users. Additionally, given the association between anemia and malaria, interventions to prevent malaria should be enhanced.

## Background

Anemia is a significant public health problem affecting around 1.93 billion people worldwide [1]. It affects 29.4% of Women of Reproductive Age (WRA) and 38.2% of pregnant women [2]. Anemia among pregnant women is associated with increased risks of maternal mortality, preterm birth, perinatal mortality and neonatal mortality, as well as low birth weight and anemia for the child [3–6]. Anemia affects cognitive development in children and reduces women’s work abilities, and it is ultimately associated with increased healthcare expenditures [2, 7, 8].

Anemia disproportionally affects developing countries, with Asian and Sub-Saharan African countries bearing 89% of the anemia burden [1]. More than 38% of all WRA in the African region are anemic [2], with significant variations between countries; in some African countries, the prevalence of anemia among WRA is higher than 50% [9].

Rwanda has made progress in improving maternal health, but anemia among WRA remains a burden. The 2014–15 Demographic and Health Survey (DHS) report estimated the prevalence of anemia among WRA in Rwanda to be 19.2% [10]. Although this is considered a mild public health problem according to WHO criteria [11], the prevalence had risen from 17% in 2010 [10]. Moreover, 11 of 30 districts in Rwanda reported much higher prevalence than the national average; with some districts even reporting prevalence of more than 30% [10, 12, 13].

The increase in national prevalence and disparity across subgroups and regions suggests that, if unaddressed, the problem will grow despite current efforts made to improve the health of the population. Understanding the risk factors for anemia is crucial for the development of innovative and evidence-based interventions to reduce its prevalence nationwide. However, the few studies conducted in Rwanda to identify risk factors for anemia were restricted to children or only a subset of WRA such as women living with Human Immunodeficiency Virus infection or young women (18–27 years old); such studies lacked national representation and also had inconsistent and conflicting results [13–18]. Accordingly, this study was conducted to understand the variations of anemia among WRA in Rwanda according to their background characteristics and to identify associated risk factors using nationally representative Rwanda Demographic and Health Survey (RDHS) data in order help inform the design of specific interventions to reduce the prevalence of anemia among WRA.

## Methods

### Study setting

Rwanda is a land-locked African country with an estimated population of 10,515,973 in 2012, of which 51.8% were female. Around 48.55% of all female are women of reproductive age, between 15 and 49 years of age [19]. The country is characterized by rapid population growth (2.6% annually) and a GDP per capita of 702.84 USD [19, 20]. Rwanda has demonstrated marked improvement in maternal health over the past 20 years [21]. In 2015, the Total Fertility Rate was 4.2 (down from 6.1 in 2005), and the maternal mortality rate was 210 per 100,000 live births, reduced from 1071 in 2000. Ninety-nine percent (99%) of pregnant women attend at least one antenatal care session, and 91% delivered at health facilities. The contraceptive prevalence rate was estimated to be 53% [10].

### Study design

This study used a quantitative, cross-sectional design by analysing data from the 2014–2015 Rwanda Demographic and Health Survey (RDHS).

### Sampling and data collection method

The 2014–15 RDHS data were collected using standard Demographic and Health Survey (DHS) questionnaires, which were adapted by stakeholders from the Rwandan government and its partners to reflect specific social and cultural issues in Rwanda. All questionnaires were translated from English to Kinyarwanda and were pre-tested prior to actual data collection. The data collection was conducted by qualified and trained professionals with rigorous supervision. Sampling was based on the 2012 Rwanda Population and Housing Census (RPHC) which consists of a list of villages, considered enumeration areas (EAs). They were stratified by type of residence (rural or urban) in each district, and 60 sampling strata were created from all districts. A two-stage cluster sampling design was used to ensure that estimates were representative at national level. At the first stage, 492 EAs were selected from all sampling strata (113 from urban areas and 379 from rural areas), and then, a systematic sampling strategy was carried out by first listing all households in selected EAs. At the second stage, 26 households were randomly selected from each EA, resulting in 12,792 households total. Anemia was tested for in half of the households selected for the general survey, resulting in a representative sample of 6680 eligible women being tested [10]. On-site hemoglobin analysis was conducted to test for anemia using a battery-operated portable HemoCue analyzer, and results were adjusted for altitude and for smoking status (if known) using the recommended Centers for Disease Control and Prevention (CDC) formula [10]. Our study analyzed all WRA in RDHS with anemia test results.

### Variables

#### Dependent variables

Our dependent variable was anemia status at the time of the survey. Pregnant women with hemoglobin level equal to or below 10.9 g/dl and non-pregnant women with hemoglobin level equal to or below 11.9 g/dl were considered anemic. The other anemia operational definitions considered were mild anemia, defined by hemoglobin levels between 10.0–10.9 g/dl and 10.0–11.9 g/dl in pregnant women and non-pregnant women, respectively; moderate anemia, defined by hemoglobin level between 7.0 and 9.9 g/dl; and severe anemia, defined by hemoglobin level less than 7.0 g/dl.

#### Independent variables

Based on previous literature and biological knowledge, the independent variables included in the analysis were social and demographic characteristics (age of the respondent, province of residence, rural vs. urban residence, educational attainment, economic status, union/marital status), variables related to reproductive health and mother’s health status (pregnancy status, number of children ever born from the woman, breastfeeding status, body mass index, use of family planning method). We also included variables related to access to information (frequency of reading newspaper or magazine, listening to radio, and watching television), and variables related to living conditions (including having a toilet facility in the household, main source of drinking water for the household, having a mosquito bed net for sleeping, respondent having slept under a mosquito bed net the night before the survey, and respondent considering distance to health facility as a problem).

### Data access and analysis

The RDHS data were downloaded from the DHS program website in STATA format after the investigators received approval from the DHS program. The “Severe”, “Moderate” and “Mild” anemia levels from the DHS was recoded into “Anemia” in our analysis; and the variable “Marital status” was regrouped into 3 levels as “Single”, “Married/living together” and “Separated/widowed”. Socio-economic status was consolidated from five to three categories. Other variables including contraceptive method, number of children ever born, and recent births in past years were regrouped by combining the less frequent options into one group for analysis. BMI was converted from a numeric to a categorical variable based on WHO body mass index [22]. We conducted descriptive analyses to summarize anemia status according to the independent variables. Pearson’s chi-squared test was used to evaluate the association between anemia status and other independent variables. To identify factors associated with anemia among WRA in Rwanda, all variables found significant in bivariate analyses were further analyzed using multivariate analysis with backward stepwise logistic regression, after checking for collinearity. A variable was removed from the full model when it was not statistically significant at *p* = 0.05. Sampling weights were included in all analyses to adjust for the effects of the stratification and cluster sampling approaches used in RDHS. Odds Ratios (OR), 95% confidence intervals (CI) and *p*-values were reported. All data analyses were conducted using STATA version 14.2 (StataCorp Lakeway Drive College Station, Texas).

## Results

### Socio-demographic characteristics

A total of 6680 women were included in the analysis. The mean age was 28.6 years (SD: 9.4). Among the samples, 1386 (20.7%) were 15–19 years old, 1225 (18.3%) were 20–24 years old, and 1153 (17.3%) were 25–29 years old. Around 24.6% (1646) of the participants were from the Eastern province, 21.6% (1442) were from the Western province, 24% (1605) were from the Southern province, 16.3% (1088) were from the Northern province and 13.5% (899) were from Kigali city. Only 184 (2.8%) had completed education above secondary level and 2863 (42.9%) completed primary school level. More than half (*n* = 3434, 51.4%) of the women were married or living together with their husbands; 2622 (39.3%) were in the poor economic category, 1249 (18.7%) were in the middle category and 2809 (42.1%) were in the rich category (Tables 1 and 2).

**Table 1:** Description of socio demographic characteristics of study participants

Variables		*n*	%
Age categories	15–19	1386	20.7
	20–24	1225	18.3
	25–29	1153	17.3
	30–34	1024	15.3
	35–39	796	11.9
	40–44	614	9.2
	45–49	482	7.2
Province	Kigali city	899	13.5
	South	1605	24.0
	West	1442	21.6
	North	1088	16.3
	East	1646	24.6
Educational attainment	Complete primary	1452	21.7
	Incomplete primary	2863	42.9
	Incomplete secondary	1044	15.6
	Complete secondary	340	5.1
	Higher	184	2.8
	No education	797	11.9
Economic Status	Poor	2622	39.3
	Middle class	1249	18.7
	Rich	2809	42.1
Marital status	Single	2543	38.1
	Married/living together	3434	51.4
	Separated/widowed	703	10.5
Type of residence	Urban	1325	19.8
	Rural	5355	80.2

**Table 2:** Description of women’ health status and living conditions of study participants

Variables		*n*	%
Currently pregnant	No or unsure	6189	92.6
	Yes	491	7.4
Number of children ever born	Had no child	2327	34.8
	1–3 children	2532	37.9
	4–6 children	1354	20.3
	7 or more	467	7.0
Currently breastfeeding	No	4787	71.7
	Yes	1893	28.3
Type of contraceptive method used	None, natural, barriers or permanent	4572	68.4
	Hormonal	1565	23.4
	Intrauterine device (IUD)	52	0.8
	Pregnant	491	7.4
Nutrition status/Body Mass Index	Normal	4774	71.6
	Underweight	411	6.2
	Overweight	1229	18.4
	Obese	258	3.9
Frequency of reading newspaper or magazine	Not at all	4916	73.7
	Less than once a week	1351	20.3
	At least once a week	403	6.0
Frequency of listening to radio	Not at all	1079	16.2
	Less than once a week	1490	22.3
	At least once a week	4099	61.5
Frequency of watching television	Not at all	3799	57.0
	Less than once a week	1760	26.4
	At least once a week	1107	16.6
Have mosquito bed net for sleeping	No	1049	15.7
	Yes	5631	84.3
Respondent slept under mosquito net	No	2184	32.7
	Yes	4496	67.3
Type of toilet facility	Nonimproved	1777	27.1
	Improved	4783	72.9
Source of drinking water	Nonimproved	1677	25.5
	Improved	4891	74.5
Access to health facility/distance	A big problem	1428	21.4
	Not a big problem	5252	78.6

### Anemia status

The mean Haemoglobin (Hb) level across the sample, adjusted for altitude and smoking status, was 13.02 g/dl (SD: 1.52), and the overall prevalence of anemia was 19.2% (95% CI: 18.0–20.5). Most cases were mild anemia, with a prevalence of 15.7% (95% CI: 14.6–16.8); 3.4% (95% CI: 2.9–3.9) were moderate anemia, and 0.2% (95% CI: 0.1–0.3) were severe anemia.

The anemia prevalence was found to be higher among women aged 45–49 years (24.2, 95% CI: 20.3–28.1) and 40–44 years (19.7, 95% CI; 16.7–23.2). It was also higher in the Southern province (22.9, 95% CI: 20.4–25.6) and in the Eastern province (21.8, 95% CI: 18.9–25.0). It was higher among women who were in the poor category (22.5, 95% CI; 20.6–24.5), who had no education (22.5, 95%CI: 19.5–25.8), who were separated from their husbands or widowed (24.8, 95% CI: 21.4–28.5), who were underweight (26.3, 95% CI: 22.0–31.1), who did not sleep under a mosquito net (21.5, 95% CI: 19.5–23.6), and who were using an intrauterine device (IUD) as a contraceptive method (29.1, 95% CI: 19.2–41.5). Anemia prevalence was relatively low among those who had completed secondary school (15.9, 95% CI: 12.1–20.7) and primary school (16.8, 95% CI: 14.8–19.1), as well as among women in the rich category (16.4, 95% CI: 14.9–18.0). See Tables 3 and 4.

**Table 3:** Prevalence of anemia per socio demographic characteristics

Variables		Anemia prevalence			*P* value
		n	%	95% CI	
Age categories	15–19	260	18.8	[16.6–21.2]	0.168
	20–24	240	19.6	[17.3–22.1]	
	25–29	208	18.1	[15.9–20.5]	
	30–34	184	18.0	[15.6–20.7]	
	35–39	155	19.5	[16.6–22.6]	
	40–44	121	19.7	[16.7–23.2]	
	45–49	116	24.0	[20.3–28.1]	
Province	Kigali city	133	14.8	[12.2–17.8]	< 0.001*
	South	367	22.9	[20.4–25.6]	
	West	258	17.9	[15.6–20.5]	
	North	167	15.4	[13.1–17.9]	
	East	359	21.8	[18.9–25.0]	
Educational attainment	Complete primary	244	16.8	[14.8–19.1]	0.010*
	Incomplete primary	580	20.3	[18.5–22.1]	
	Incomplete secondary	189	18.1	[15.8–20.6]	
	Complete secondary	54	15.9	[12.1–20.7]	
	Higher	38	20.7	[15.6–26.9]	
	No education	179	22.5	[19.5–25.8]	
Economic Status	Poor	589	22.5	[20.6–24.5]	< 0.001*
	Middle class	235	18.8	[16.3–21.7]	
	Rich	460	16.4	[14.9–18.0]	
Marital status	Single	485	19.1	[17.4–20.8]	0.001*
	Married/living together	625	18.2	[16.7–19.8]	
	Separated/widowed	174	24.8	[21.4–28.5]	
Type of residence	Urban	217	16.3	[14.3–18.6]	0.010*
	Rural	1068	19.9	[18.5–21.5]	

*P* value are from Pearson’s chi-squared test

*: Unadjusted significant variables (*p* < 0.05)

**Table 4:** Prevalence of anemia per women’ health status and living conditions

Variables		Anemia prevalence			*P* value
		n	%	95% CI	
Currently pregnant	No or unsure	1170	18.9	[17.7–20.2]	0.019*
	Yes	115	23.4	[19.6–27.6]	
Number of children ever born	Had no child	456	19.6	[17.9–21.4]	0.684
	1–3 children	472	18.6	[17.0–20.4]	
	4–6 children	259	19.2	[17.0–21.6]	
	7 or more	97	20.8	[17.1–25.2]	
Currently breastfeeding	No	919	19.2	[17.8–20.6]	0.918
	Yes	366	19.3	[17.4–21.4]	
Type of contraceptive method used	None, natural, barriers or permanent	940	20.6	[19.1–22.1]	< 0.001*
	Hormonal	215	13.7	[11.9–15.8]	
	Intrauterine device (IUD)	15	29.1	[19.2–41.5]	
	Pregnant	115	23.4	[19.6–27.6]	
Nutrition status/Body Mass Index	Normal	912	19.1	[17.8–20.5]	< 0.001*
	Underweight	108	26.3	[22.0–31.1]	
	Overweight	233	19.0	[16.7–21.5]	
	Obese	30	11.6	[8.1–16.4]	
Frequency of reading newspaper or magazine	Not at all	970	19.7	[18.3–21.2]	0.199
	Less than once a week	235	17.4	[15.4–19.6]	
	At least once a week	78	19.3	[15.5–23.8]	
Frequency of listening to radio	Not at all	233	21.6	[18.8–24.7]	0.138
	Less than once a week	292	19.6	[17.2–22.1]	
	At least once a week	759	18.5	[17.1–20.0]	
Frequency of watching television	Not at all	755	19.9	[18.3–21.5]	0.237
	Less than once a week	330	18.8	[16.8–20.9]	
	At least once a week	195	17.6	[15.3–20.1]	
Have mosquito bed net for sleeping	No	232	22.1	[19.4–25.1]	0.020*
	Yes	1052	18.7	[17.4–20.1]	
Respondent slept under mosquito net	No	470	21.5	[19.5–23.6]	0.003*
	Yes	815	18.1	[16.8–19.6]	
Type of toilet facility	Nonimproved	391	22.0	[19.7–24.4]	0.003*
	Improved	871	18.2	[16.9–19.6]	
Source of drinking water	Nonimproved	327	19.5	[17.0–22.3]	0.785
	Improved	935	19.1	[17.8–20.5]	
Access to health facility/ distance	A big problem	285	20.0	[17.5–22.7]	0.503
	Not a big problem	999	19.0	[17.7–20.4]	

*P* values are from Pearson’s chi-squared test

*: Unadjusted significant variables (*p* < 0.05)

The bivariate analysis found that 11 variables were significantly associated with anemia: 1) province of residence (*p* < 0.001), 2) educational attainment (*p* = 0.010), 3) economic status (p = < 0.001), 4) marital status (*p* = 0.001), 5) pregnancy status (*p* = 0.019), 6) type of FP method used (*p* = < 0.001), 7) nutrition status (p < 0.001), 8) having a mosquito bed net for sleeping (*p* = 0.020), 9) having slept under a mosquito bed net the night before the survey (*p* = 0.003), 10) type of toilet facility used in the household (*p* = 0.003), and 11) type of residence (*p* = 0.010) See Tables 3 and 4.

### Factors associated with anemia

Six risk factors for anemia among WRA were highlighted in the multivariate analysis: 1) nutrition status, 2) economic status, 3) type of contraceptive method used, 4) use of a mosquito net, 5) marital status, and 6) province of residence (Table 5).

**Table 5:** Factors associated with anemia: multivariate analysis results (Logistic Regression)

Variables	Full model				Final model		
	AOR	95% CI	P-value		AOR	95% CI	P-value
Age categories							
15–19	1						
20–24	1.13	[0.90–1.42]	0.285				
25–29	1.01	[0.77–1.33]	0.915				
30–34	1.02	[0.76–1.37]	0.871				
35–39	1.18	[0.86–1.60]	0.304				
40–44	1.07	[0.77–1.49]	0.669				
45–49	1.24	[0.88–1.75]	0.223				
Nutrition status/Body Mass Index							
Normal	1				1		
Underweight	1.38^*^	[1.08–1.78]	0.011		1.39*	[1.09–1.77]	0.009
Overweight	1.1	[0.93–1.31]	0.270		1.09	[0.92–1.30]	0.301
Obese	0.60^*^	[0.40–0.91]	0.015		0.61*	[0.41–0.92]	0.019
Economic Status							
Poor	1				1		
Middle class	0.83	[0.69–1.01]	0.057		0.83*	[0.69–1.00]	0.044
Rich	0.74^**^	[0.61–0.89]	0.002		0.74*	[0.63–0.87]	< 0.001
Type of contraceptive method used							
None - natural - barriers or permanent	1				1		
Hormonal	0.60^*^	[0.49–0.73]	< 0.001		0.60^*^	[0.50–0.72]	< 0.001
IUD	1.93^*^	[1.01–3.68]	0.045		1.97^*^	[1.04–3.73]	0.037
Pregnant	1.18	[0.90–1.53]	0.227		1.15	[0.89–1.49]	0.275
Educational attainment							
No education	1						
Incomplete primary	0.97	[0.78–1.21]	0.810				
Complete primary	0.81	[0.64–1.03]	0.087				
Incomplete secondary	0.99	[0.74–1.32]	0.929				
Complete secondary	0.89	[0.61–1.30]	0.545				
Higher	1.35	[0.88–2.08]	0.171				
Respondent slept under mosquito bed net							
No	1				1		
Yes	0.85^*^	[0.74–0.98]	0.031		0.85^*^	[0.73–0.98]	0.025
Marital status							
Single	1				1		
Married/living together	1.07	[0.86–1.34]	0.537		1.11	[0.94–1.30]	0.215
Separated/widowed	1.28	[0.98–1.69]	0.073		1.35^*^	[1.09–1.67]	0.006
Type of toilet facility							
Nonimproved	1						
Improved	0.96	[0.82–1.12]	0.624				
Type of residence							
Urban	1						
Rural	0.97	[0.79–1.18]	0.745				
Province of residence							
Kigali city	1				1		
South	1.52^*^	[1.17–1.97]	0.002		1.45^*^	[1.15–1.82]	0.002
West	1.07	[0.81–1.41]	0.636		1.03	[0.80–1.33]	0.814
North	0.95	[0.71–1.28]	0.756		0.92	[0.70–1.20]	0.533
East	1.50^*^	[1.15–1.95]	0.003		1.41^*^	[1.11–1.79]	0.005

*: Adjusted significant variables (*p* < 0.05) from multiple logistic regression

AOR: Adjusted Odds Ratio.

Compared to WRA with normal BMI, WRA who were underweight were 1.39 times as likely to have anemia (OR: 1.39, 95% CI: 1.09–1.77, *p* = 0.009) while obese women were 0.61 times as likely to have anemia (OR: 0.61, 95% CI: 0.41–0.92, *p* = 0.019).

Compared to women in the poor category, women in the rich category were 0.74 times as likely to have anemia (OR: 0.74, 95% CI: 0.63–0.87, *p* < 0.001) and women in the middle category were 0.83 times as likely (OR: 0.83, 95% CI: 0.69–1.00, *p*-value = 0.044) to have anemia.

Women who were using hormonal contraceptives were 0.6 times as likely to have anemia (OR: 0.60, 95% CI: 0.50–0.72, p-value: < 0.001) while those who were using intrauterine devices were 1.97 times as likely to have anemia (OR: 1.97, 95% CI: 1.04–3.73, p-value = 0.037) compared with those using natural barriers, permanent contraceptive methods, or no contraceptive method. Compared to women who were not married, women who were separated or widowed were 1.35 times as likely to have anemia (OR: 1.35, 95% CI: 1.09–1.67, *p* = 0.006). There was no difference between married and non-married women (*p* = 0.25).

Women who reported sleeping under a mosquito net the night before the survey were 0.85 times as likely to have anemia (OR: 0.85, 95% CI: 0.73–0.98, *p* = 0.025) than those who did not sleep under a mosquito net the night before the survey. Additionally, women who were living in Southern province (OR: 1.45, 95% CI: 1.15–1.82, *p* = 0.002) and in Eastern province (OR: 1.41, 95% CI: 1.11–1.79, *p* = 0.005) were 1.45 and 1.41 times as likely to have anemia, respectively compared to WRA living in Kigali city.

## Discussion

The objectives of this study were to identify the describes of the background characteristics of WRA in Rwanda with anemia and to therefore identify associated risk factors. According to the analysis, the prevalence of anemia among WRA in Rwanda was 19.2%. Although the anemia prevalence in Rwanda was lower than many other countries in the Sub-Saharan Africa region [2, 9], it is still considered a public health problem according to WHO criteria [23]. In addition, the prevalence increased between 2010 and 2015 and varied across population subgroups [10, 24]. In this study, it was found to vary with age, province of residence, education level, marital status, the type of contraceptive method used as well as the economic and nutrition status. Our study showed that women who were obese or rich, as well as those who slept under a mosquito net or used hormonal contraceptives were less likely to have anemia while those who were underweight, used intrauterine devices as a contraceptive method, and lived in the Southern or Eastern provinces were more likely to have anemia, than were individuals in their respective comparison groups.

Similar to studies in other settings including Ethiopia and Pakistan, our analysis found that poor and undernourished women were more likely to have anemia [25, 26]. Anemia is a multifaceted problem where nutrition and economic status work in synergy. Evidence suggests that improved economic status is associated with appropriate nutrition conditions [27], lower infection morbidity [26], increased access to health services as well as other favourable living conditions [27, 28], all of which in turn influence anemia. Malnourished women have greater risk of iron deficiency, the most common proximate cause of anemia [1] and malnutrition is often associated with poor socio-economic status [29]. Interventions that aim to empower women economically should be considered in order to reduce anemia prevalence. Moreover, malnutrition management programs should ensure that iron supplementation is sustained within intervention packages.

In this study, the use of hormonal contraceptives was associated with lower risk of anemia among WRA, while the use of IUD was associated with higher risk. Similar findings were seen in other studies conducted in 14 different low- and middle-income countries including Tanzania and Ethiopia [25, 30, 31]. Another study conducted in seven countries also found that hormonal contraceptive users had higher haemoglobin and ferritin levels compared to non-users [32]. Using hormonal contraceptive can be resulted in less bleeding during the menstruation, which ultimately reduces blood loss over time [33, 34].

A study conducted in Pakistan also observed higher anemia risk among IUD users [35]. IUD may increase uterine blood flow as well as volume of bleeding during and duration of menstruation periods, especially during the first months of usage, which in turn increase the likelihood of anemia [36, 37]. In addition, some research has found that IUD users have a reduction in hemoglobin content and iron saturation/ ferritin levels, which may trigger or worsen existing anemia [32, 38]. While more investigations are needed to understand the real physiological mechanisms, our study findings supported the existing evidence that IUD use is among the risk factors of anemia in WRA. Clinical guidelines should consider specifying treatments for IUD-induced bleeding [39] as well recommending iron supplementation for IUD users especially during the first months of usage.

Geographic area of residence was found to be associated with anemia, with women in the Eastern and Southern provinces being more likely to have anemia. The Eastern and Southern provinces in Rwanda are considered to be high malaria endemic regions; higher risk of malaria also translates to higher anemia risk [40]. Similar associations between anemia and geographic location have been found in Tanzania [30]. In Rwanda, iron supplementation during pregnancy is less common in the Eastern province than in other provinces [10]. Interventions should consider including iron supplementation, promotion of foods rich in iron and other micronutrients, as well as to prevent malaria [12, 41]. The most affected geographic areas should be prioritized.

Consistent with the results from other studies, sleeping under mosquito nets was associated with lower likelihood of anemia in our study, which makes sense given that malaria itself is a risk factor of anemia [42]. As mosquito net coverage and usage remain challenges in many developing countries [43–45], malaria prevention strategies including efforts to ensure the availability as well as proper use of mosquito nets in the community should be integrated in anemia prevention programs.

Widowers or women separated from their husbands were more likely to have anemia. Traditionally men are breadwinners in many developing societies [46]. Widows and women separated from their husbands lack support to sustain their families, predisposing them to economic deprivation, poverty, malnutrition and low access to health services [27–29]. In addition, our analysis showed a correlation between marital status and age (r = 0.63). Older age was found to be associated with anemia in some studies [25]. While further investigations are needed to better understand the possible associations between marital status, age and anemia status, our findings suggest that old women, especially widows, may face many other health problems that are understudied. Special attention and priority should be given to understanding the health needs of this vulnerable group.

Our study found that women with lower education levels had slightly higher prevalence of anemia, although statistical significance was not found. Other studies have found education level to be a risk factor for anemia [25, 30]. The differences in settings of the studies could be related to the discrepancy. In the 2014/2015 Rwanda DHS, only 19% of women had no education; while 67% were reported in Ethiopia in 2005 and 27% in Tanzania in 2010 [10, 47, 48], the variation in the sample composition could have affected the analysis outcome.

Interestingly, this study found that about 40% of the women classified as “rich” also had anemia. This result was inconsistent with other evidence. Despite economic improvement in Rwanda over recent decades, about 39% of the population remained living under poverty line [49, 50]. Further investigation found that DHS, the Ministry of Local Government and Social Affairs in Rwanda used different socioeconomic classification methodologies. The DHS used five categories: poorest, poor, middle, rich and richest, based on the durable goods owned by a household such as television, mobile telephone and other household characteristics such as access to electricity and source of drinking water [10], while the Rwanda Ministry of Local Government and Social Affairs uses four categories for social stratification: very poor, poor, middle and rich [51, 52]. In recent years, there has been considerable debate over whether the stratification systems truly reflect the economic status of the population [53, 54]. Acknowledging that the limitations of the stratifications may cause discrepancies, the Rwanda government is currently revising the categorization to reflect the true population economic status [52].

This study successfully identified some risk factors among WRA in Rwanda and proposed some recommendations. However, the results must be seen in light of some limitations. This study could only use variables that were in the DHS, due to the nature of secondary data analysis. Qualitative information could provide increased understanding of the attitudes and practices related to variations in food consumption patterns. However, the DHS survey used a national representative sample and was conducted with standardized quality assurance measures in both data collection and management to ensure reliability and validity of the results [55, 56], which could improve the generalizability of the results of our analysis.

## Conclusions

In order to address anemia among WRA in Rwanda, programs should seek to improve women’s economic livelihoods and nutritional statuses. Furthermore, clinical guidelines should ensure that IUD users have access IUD-related bleeding treatments as well as iron supplements. Special attention should be provided based on geographic variations. Integrating malaria prevention strategies into anemia program should also be considered.

## Data Availability

The data used for this study are from the Rwanda Demographic and Health surveys (DHS) and are publicly available here: https://dhsprogram.com/data/available-datasets.cfm

## Abbreviations

AOR: Adjusted Odds Ratio
CI: Confidence Interval
DHS: Demographic and Health Survey
FP: Family Planning
Hb: Hemoglobin
OR: Odds Ratio
RDHS: Rwanda Demographic and Health Survey
WHO: World Health Organization
WRA: Women of Reproductive Age
